# Double-negative T cells are increased in HIV-infected patients under antiretroviral therapy

**DOI:** 10.1097/MD.0000000000030182

**Published:** 2022-09-09

**Authors:** Fatma Korbi, Imen Zamali, Raja Rekik, Ahlem Ben Hmid, Mouldi Hidri, Wafa Kammoun Rebai, Zeineb Jelili, Senda Masmoudi, Souleiman Karim Rahal, Azza Ben Ayed, Mélika Ben Ahmed

**Affiliations:** a Department of Clinical Immunology, Pasteur Institute of Tunis, Tunis, Tunisia; b Faculty of Medicine of Tunis, University of Tunis El Manar, 1068, Tunis, Tunisia; c Laboratory of Biomedical Genomics and Oncogenetics, Pasteur Institute of Tunis, Tunis, Tunisia; d Faculty of Pharmacy, Monastir, Tunisia.

**Keywords:** double-negative T cells, HIV, immunophenotyping, viral load

## Abstract

Double-negative T (DNT) cells are a T-cell subset with a CD4^–^CD8^–^ phenotype. They represent 1% to 5% of circulating lymphocytes, but an increase in this proportion can be found during lymphoproliferative and autoimmune diseases. This increase has also been reported in persons with HIV (PWH). The aim of this work was to better describe the proportion of DNT cell subset in PWH. We retrospectively collected 984 samples from PWH referred for lymphocyte immunophenotyping over a 7.5-year period. Quantification of DNT cells was performed by flow cytometry. DNT cell proportion was calculated by subtracting the CD4^+^ and CD8^+^ subsets proportions from the total of T cells. A total of 984 blood samples from PWH were collected. Mean CD4 T-cell count was decreased in such patients while DNT cell frequency was increased with a mean of 6.7%. More than half of the patients had a DNT cell proportion >5%. Patients with DNT cell proportion over 5% exhibited significantly reduced CD3^+^ and CD4^+^ T-cell counts, while CD8^+^ T-cell count was unchanged compared to patients with normal DNT cell rates. Interestingly, DNT cell percentage was negatively correlated with CD4 and CD3 T-cell counts in all included patients. Moreover, the DNT cell proportion was significantly increased in subjects with CD4^+^ T cells <200/mm^3^ compared to those with CD4^+^ T cells >200/mm^3^. Interestingly, DNT cell proportions were significantly higher in patients with high viral load compared with those presenting undetectable viral load. HIV infection is associated with an increase in DNT cell proportion. This increase is more frequent as the CD4 count is decreased and the viral load is increased. DNT cell subset should not be omitted when interpreting immunophenotyping in PWH as it appears to be associated with disease progression in patients under antiretroviral therapy.

## 1. Introduction

Double-negative (DN) T cells express a CD3^+^CD4^–^CD8^–^ phenotype. They are present in peripheral blood, lymph nodes, and mucosa-associated lymphoid tissue.^[1]^ Their functions and ontogeny are still poorly established. The variety of surface markers as well as the different cytokine and chemokine expression profiles of such cells point out the presence of several differentiation pathways.^[1]^

DNT cells exhibit a duality of function as they appear to have effector as well as regulatory functions. DNT cell effector functions have been suggested by rapid expansion of such cells following parasitic, viral and intracellular bacterial infection, as well as the expression of an array of proinflammatory effector cytokines.^[2–4]^ On the other hand, DNT cells seem to be involved in the maintenance of immune homeostasis as they are able to regulate autoimmunity and contribute to tolerance induction after transplantation.^[1,5]^

DNT cells represent 1% to 5% of circulating T cells.^[1,6]^ An increase in this rate is associated with several pathological conditions, mainly lymphoproliferative and autoimmune disorders.^[7]^ A high rate has also been reported during infections, mainly with the human immunodeficiency virus (HIV) and its precursor in nonhuman primates, Simian immunodeficiency virus (SIV).^[6]^

In this study, we proposed to evaluate DNT cell levels in persons with HIV (PWH) under antiretroviral therapy (ART) and to look for a possible correlation with other lymphocyte populations and the viral load.

## 2. Material and Methods

### 2.1. Study population

This retrospective study included 984 PWH referred to the clinical immunology laboratory of Pasteur institute of Tunis for lymphocyte immunophenotyping from January 2014 to June 2021. At the time of blood collection, patients were already on ART. The Bio-Medical Ethics Committee of Pasteur Institute of Tunis approved the study and informed consent was obtained from all participants.

### 2.2. Immunophenotyping

Freshly collected EDTA-anticoagulated whole blood was processed within 2 to 4 hours after sampling. After lysis of red blood cells and washings with PBS containing 1% BSA, cells were incubated at room temperature for 20 minutes with the following conjugated monoclonal antibodies from BD Biosciences: anti-CD45-allophycocyanin-H7, CD4-fluorescein isothiocyanate, anti-CD8-phycoerythrin, and CD3-phycoerythrin-cyanin-5. Cells were fixed with Cell Fix (BD Biosciences) and kept at 4°C until analysis with a BD FACSCanto™ II flow cytometer. DNT cell proportions were determined by subtracting the CD4^+^ and CD8^+^ T-cell subsets proportions from the total of T cells: CD3^+^ – (CD3^+^CD4^+^ + CD3^+^CD8^+^). For each sample, a complete blood count was performed as well to assess total lymphocyte count and calculate the different T-cell subset counts.

### 2.3. Viral load measurement

Plasma HIV-1 viral load was quantified by real-time PCR. The sensitivity of viral RNA detection was 40 copies/mL of plasma.

### 2.4. Statistical analysis

Statistical analyses were performed using SPSS (Version 22, IBM^®^, USA). The difference between the groups was determined using the nonparametric Mann–Whitney *U* test. Correlation between different T-cell subset proportions or counts were calculated with the Spearman Rank correlation coefficient. *P* values <.05 were considered statistically significant.

## 3. Results

A total of 984 blood samples were collected from HIV-infected patients with a sex ratio of 1.16 and an average age of 36 ± 14.9 years. The different mean T-cell subset counts and proportions are shown in **Table 1**. Fifty-one percent of patients (500/984) had a CD4 T-cell count <500/mm^3^ and 45% (443/984) had a CD8 T-cell count >900/mm^3^. Half PWH (51.7%; 509/984) had a DNT cell proportion >5% and 15.8% (155/984) of them had a proportion >10%.

**Table 1:** Mean counts and proportions of the different T-cell subsets.

Parameter	Mean	Reference range
White blood cells	5900/mm^3^ (840–16,300)	4000–10,000/mm^3^
Lymphocytes	1920/mm^3^ (66–8890)	1500–4000/mm^3^
T cells %	83.1% (14–97)	60%–85%
T-cell count	1593/mm^3^ (24–8287)	850–2200/mm^3^
CD4 T cells %	25.3% (0–85)	35–65%
CD4 T-cell count	487.1/mm^3^ (0–4672)	500–1400/mm^3^
CD8 T cells %	47.6% (2.2–95.2)	13%–40%
CD8 T-cell count	836/mm^3^ (0–3850)	200–900/mm^3^
CD4/CD8 ratio	0.53 (0–8.95)	1.0–3.0
DNT cell %	6.7% (0–27)	1%–5 %
DNT cell count	98.67/mm^3^ (0–1041)	

DN = double negative.

**Figure 1.**
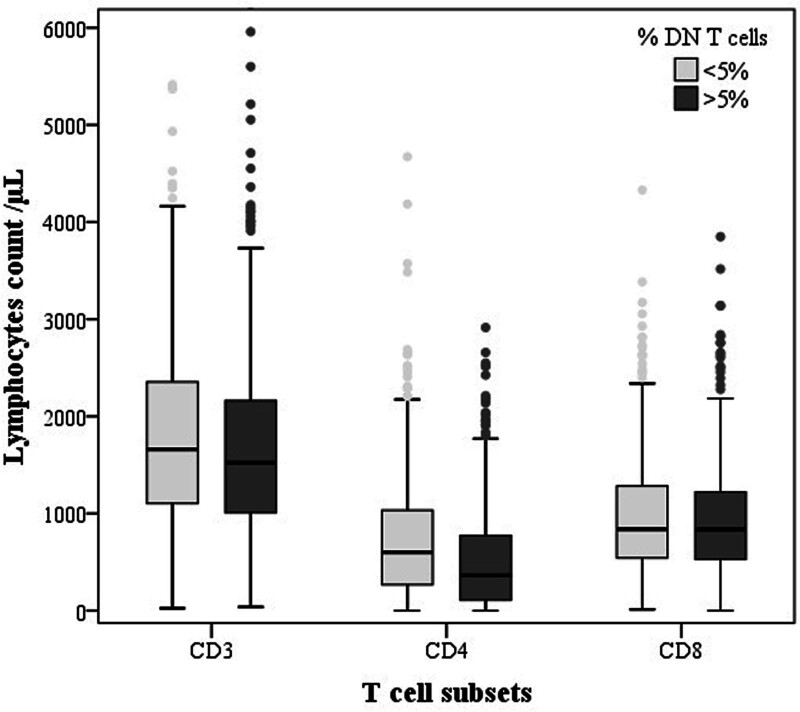
Mean of CD3, CD4, and CD8 T-cell subset counts in patients with DNT cell proportion within normal range (<5%) and in patients with increased DNT cell proportion (>5%). DNT = double negative T.

When comparing different T-cell subset levels in patients presenting a DNT cell proportion >5% to those with DNT cell proportions within normal range, we noted significantly reduced CD3^+^ (1521 vs 1660.5/mm3, *P* = .016) and CD4^+^ (363 vs 601/mm^3^, *P* < .001) T-cell counts while CD8^+^ T-cell count was similar (836 vs 837.9/mm^3^, *P* = .49; **Fig. 1**). Subsequently, CD4/CD8 ratio was statistically decreased in the former group of patients (0.4 vs 0.69; *P* < .001). Besides, lymphocyte count was also lower in these patients (1859 vs 2001/mm^3^; *P* = .017).

**Figure 2.**
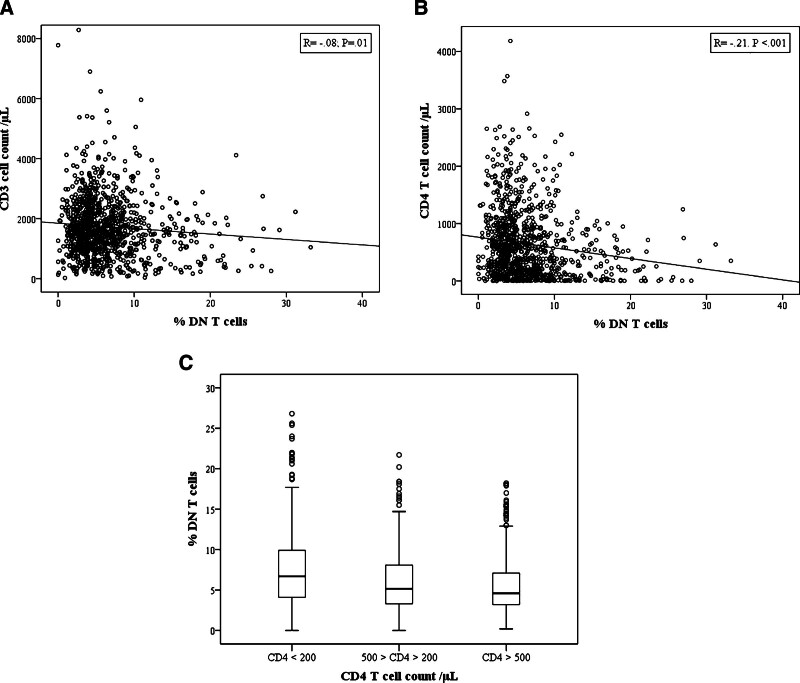
(A) Correlation of DNT cell proportion with CD3 T-cell counts. (B) Correlation of DNT cell proportion with CD3 T-cell counts. The Spearman rank test was used for correlation analysis. (C) Mean of DNT cell proportion according to CD4 T-cell count. ns: nonsignificant. DNT = double negative T.

We analyzed the relationship between T-cell subset counts and DNT cell proportion and observed a negative correlation with CD3 and CD4 T-cell counts (**Fig. 2A**, B) and no significant correlation with CD8 T-cell count (*P* = .16). Interestingly, the DNT cell proportion was significantly increased in subjects with CD4^+^ T cells < 200/mm^3^ compared to those with CD4^+^ T cells >200/mm^3^ (8.3% vs 5.7%, *P* < .001; **Fig. 2C**).

**Figure 3.**
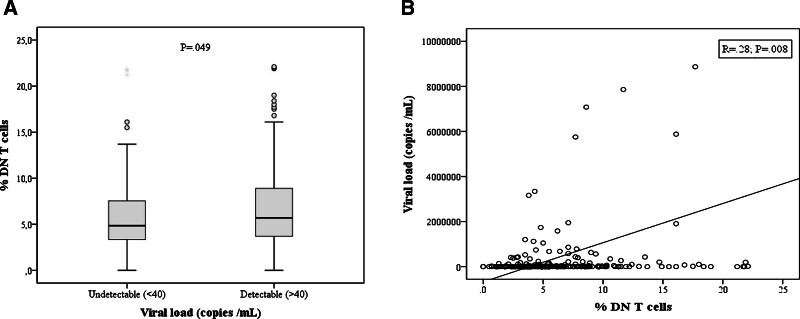
(A) Mean of DNT cell proportions in patients with undetectable and detectable viral load. (B) Correlation of DNT cell proportion with the viral load. The Spearman rank test was used for correlation analysis. DNT = double negative T.

The viral load was available at the time of lymphocyte immunophenotyping in 221 cases. Among these, 117 (52.94%) patients had a DNT cell proportion above 5%, and 37 (16.74%) patients above 10%. Ninety-six patients had undetectable viral load with a median CD4 T-cell count of 603/mm^3^ and median DNT cell proportion of 4.85%. In patients with detectable viral load (n = 125, median viral load = 33884), the median CD4 T-cell count was 271/mm^3^ and their median DNT cell proportion was 5.7%, which was higher than that in patients with undetectable viral load (*P* = .049; Fig. 3A). DNT cell proportion was positively correlated with the viral load (*P* = .008; Fig. 3B). Furthermore, the viral load was significantly higher in patients with DNT cell proportion >5% compared to those within the normal range (*P* = .015; data not shown).

## 4. Discussion

Our study showed a CD4 T-cell number near the lower limit, and a slightly increased CD8 T-cell count in PWH with a clearly inverted CD4/CD8 ratio. These variations are frequent in the early stages of infection or in the AIDS stage. Less commonly reported, DNT cell proportion was increased in nearly half of the patients and was negatively correlated with CD3 and CD4 T-cell counts. Interestingly, increase in DNT cell proportion was more pronounced in subjects with CD4^+^ T cells <200/mm^3^. Although Liang et al^[8]^ showed that DNT cell levels decrease as the disease progressed in ART-naive PWH, ART was reported to be able to restore DNT cell levels in PWH whether they revealed to be responders or not.^[9]^ That may explain the increased DNT cell frequency in our patients, all of which were under ART.

The significant positive correlation between DNT cells and the viral load observed in our cohort was consistent with the negative correlation found between DNT cells and CD4 T-cell count. Indeed, patients with an undetectable viral load and a restored CD4 T-cell count under ART had a lower proportion of DNT cells than patients with a detectable viral load and a low CD4 T-cell count. Previous studies have reported different results from ours. The correlation between DNT cells and viral load was either negative or absent in untreated patients.^[8–10]^ No study has yet examined this association in treated patients. Nevertheless, considering the correlation between CD4 and DNT cells, our results align with those of Singleterry et al.^[11]^ Their study demonstrated an increase of DNT cells in 31 PWH who had advanced to the AIDS stage of infection, and this expansion increased as the CD4 count lowered.^[11]^ Our results confirm these data on a larger cohort.

The increase in DNT cells in PWH may reflect a systemic immune response to the CD4 T-cell depletion and thus would be of central thymic origin. A second hypothesis could be that DNT cells have a peripheral origin due to the loss of CD4 expression. HIV-1 gene products such as Nef, Env, and Vpu have been involved in CD4 down-modulation.^[12,13]^ Further studies suggest that it can also occur independently of viral proteins, implicating rather an atypical regulation of the DNA methylation machinery leading to epigenetic silencing of CD4 expression.^[14]^

DNT cells seem essential in compensating CD4 T-cell depletion as they are associated to the nonpathogenic nature of the infection in SIV-infected natural hosts.^[15]^ Natural hosts, such as sooty mangabeys and African green monkeys, do not develop AIDS and are able to maintain preserved T-cell populations and low levels of systemic immune activation despite high levels of virus replication.^[16,17]^ These primates exhibit up to 40% of DNT cells that are capable of proliferating and producing T helper cytokines following antigen stimulation.^[6,15]^

Transforming growth factor-beta 1 and interleukin-10-producing DNT cells are associated with the control of chronic immune activation, which plays an essential role in the pathogenesis of HIV infection and is a key predictor of progression to AIDS.^[9,10]^ Furthermore, DNT cells seem to be involved in the immune reconstitution occurring during ART and to predict future immune activation.^[9]^ This immunoregulatory function might explain the persistence of high levels of immune activation in PWH nonresponding to long-term ART, whose DNT cell levels were shown to be only partially restored^[9]^

This study presents some limitations. Its retrospective nature leads to a lack of clinical data on the stage of the disease that would allow a better analysis of the correlation with the progression of the infection. Another limitation is that we only included PWH under ART and this does not allow us to generalize our results to all PWH. On the other hand, the strong point of this study is a large number of patients included.

## 5. Conclusion

Altogether, data from the present study show that, in addition to a decrease in CD4 and an increase in CD8 T cells, an increase in DNT cell proportion is frequently found in HIV-infected patients. It is important for the biologist and the clinician to keep in mind the possible DNT cell level variations when interpreting immunophenotyping results in PWH. The significant associations with CD4 T-cell count and the viral load strongly support that DNT cells are correlated with disease progression in treated PWH. Therefore, this marker should be considered for use in clinical practice.

## Author contributions

Conceptualization: IZ and MBA; Acquisition of data: FK, IZ, RR, ABH, ZJ, ABA, SKR, WKR and MH; Analysis and

interpretation of data: FK, IZ and MBA; Writing - Original draft preparation: FK and IZ; Final approval: MBA.
